# A Case of Palpable Purpura Associated With Japanese Spotted Fever

**DOI:** 10.7759/cureus.94711

**Published:** 2025-10-16

**Authors:** Misaki Kase, Tatsushi Ishimoto, Kozo Nakai

**Affiliations:** 1 Dermatology, Kochi University, Kochi, JPN

**Keywords:** direct immunofluorescence assay, iga vasculitis (igav), japanese spotted fever, rickettsial infection, tick bite

## Abstract

This report presents the case of a 90-year-old man with Japanese spotted fever (JSF), caused by *Rickettsia japonica*, who initially manifested high fever, rash, eschar, and lymphadenopathy. After successful antibiotic therapy, he developed palpable purpura similar to IgA vasculitis (IgAV). Previous reports indicate that the rash in spotted fever group of rickettsial infections, including JSF, undergoes a transformation from petechial spots after three to four days. However, in this case, the rash resolved completely after one week, and on day 10 of hospitalization, new purpura with palpable infiltration developed, leading to the consideration of IgAV. A skin biopsy and direct immunofluorescence assay revealed findings that suggested IgAV. IgA elevation was observed in the blood test. IgAV has been reported to be caused by various factors, including bacterial and viral infections, vaccines, and medications, and solid-organ malignancy is recognized as a risk factor. Although this patient was elderly, there were no serious underlying conditions. While there were no regular oral medications, minocycline and levofloxacin were used for rickettsiosis treatment; however, no association between these two agents and IgAV development has been reported.

This case suggests that JSF may have triggered secondary IgAV, emphasizing the need for clinicians to consider IgAV when palpable purpura develops after treatment for rickettsial infection.

## Introduction

Japanese spotted fever (JSF) is a tick-borne rickettsiosis caused by the bacterium *Rickettsia japonica*. JSF develops abruptly and is characterized by symptoms such as headache, fever, chills, and malaise. JSF skin lesions are characterized by erythematous rash and one or more eschars at the tick bite sites [1]. Some of the rashes turn into petechial spots in later stages [1]. Histopathologically, inflammation of vascular endothelial cells and leukocytoclastic vasculitis are commonly observed in both the rash and eschar lesions [2,3]. Immunoglobulin A (IgA) vasculitis (IgAV), formerly known as Henoch-Schönlein purpura, is also a type of leukocytoclastic vasculitis. It is characterized by deposition of IgA in the small vessel walls of the skin [4].

Herein, we present the case of a 90-year-old man diagnosed with JSF, in whom, following the complete disappearance of the initial rash, a new eruption of not petechial spots but palpable purpura developed, suggestive of secondary IgAV. The skin biopsy of the palpable purpura showed a histopathological finding suggestive of IgAV, and direct immunofluorescence assay (DIF) revealed IgA deposition in a small vessel corresponding to those seen in IgAV. IgA vasculitis can be provoked by various triggers, but more than 50% of cases occur following an upper respiratory tract infection [5]. However, a few reports of IgAV following rickettsial infection have been documented [6]. This case emphasizes the fact that when palpable purpura develops during treatment for JSF, clinicians should consider the possibility of secondary IgAV.

## Case presentation

**Figure 1:**
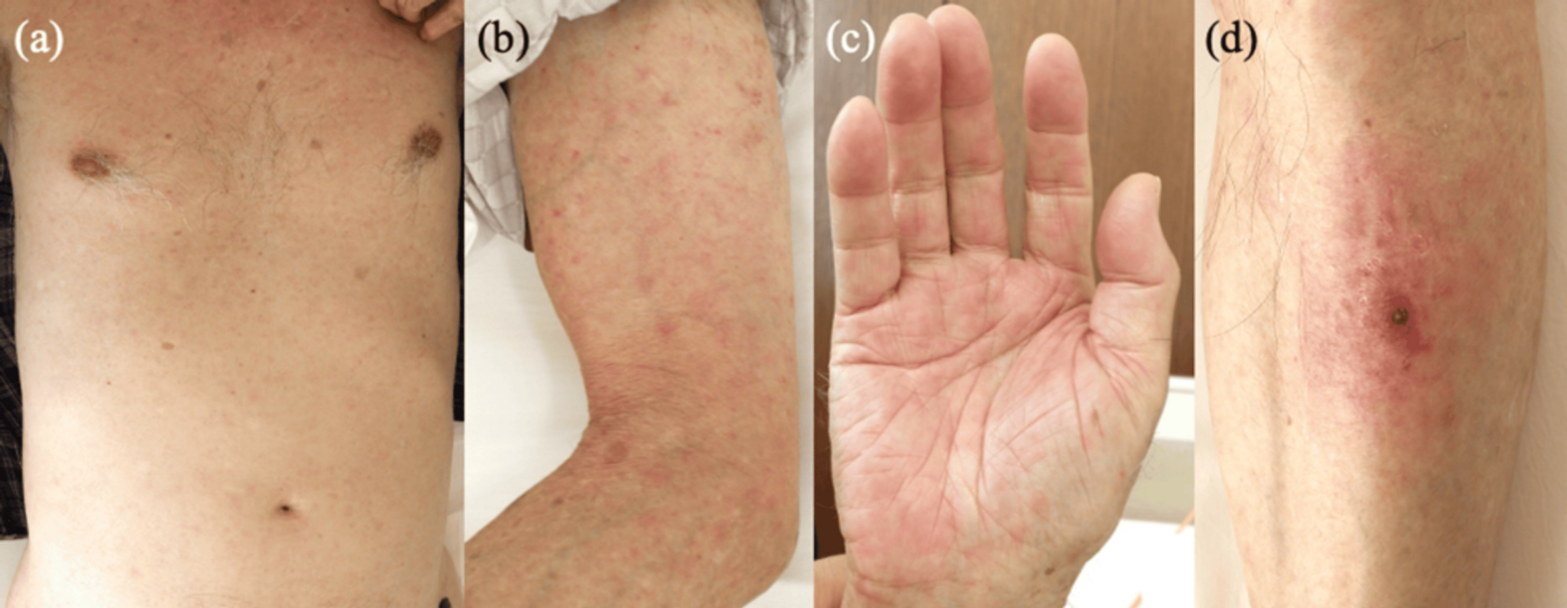
Clinical features, day 1. (a-c) Erythematous macules and papules on the trunk, extremities, and palms. (d) An eschar surrounded by erythema on the left lower leg.

A 90-year-old man living in Ino-cho, Kochi, Japan, was found by a neighbor, who noticed that he was unable to walk. He had experienced high fever, malaise, loss of appetite, and muscle pain throughout his body for the previous five days. His medical history included only insomnia, and he occasionally took Eszopiclone (1 mg/day). He was transported by ambulance to our hospital. On physical examination, in addition to generalized myalgia, erythematous rash on the trunk, extremities, and palms (Figures 1a-1c), an eschar surrounded by erythema on the left lower leg (Figure 1d), and bilateral enlarged inguinal lymph nodes were observed. Laboratory examinations showed leukocytosis (11,920 counts/μL [normal: ≤9,000 counts/μL]), thrombocytopenia (8.1×104/μL [normal: 15-35×104/μL]), and elevated liver enzymes and C-reactive protein (10.45 mg/dL [normal: <0.5 mg/dL]). The eosinophil count was deficient from day 2. These clinical manifestations strongly suggested rickettsiosis, including JSF and tsutsugamushi disease, which are prevalent in Japan [7].

**Figure 2:**
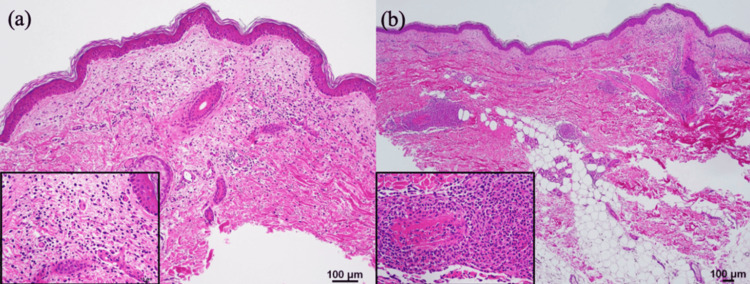
Histopathological findings of pale erythema on the right thigh, day 1. (a) Leukocyte infiltration was observed in the upper dermis, and nuclear dust presented around small blood vessels. Hematoxylin and eosin staining (original magnification ×100; left lower insert, ×400). (b) Leukocytoclastic vasculitis with granulomatous inflammation in medium-sized vessels in the middle to lower layers of the dermis. The vessel walls were damaged and fibrin leaked out, presenting as fibrinoid necrosis. Hematoxylin and eosin staining (original magnification ×40; left lower insert, x400).

Histology of the biopsy specimen from the pale erythema on the right thigh at day 1 showed leukocyte infiltration and nuclear dust in the upper dermis (Figure 2a). There was leukocytoclastic vasculitis with granulomatous inflammation in the medium-sized vessels of the middle to lower dermal layers. Damage to the vessel walls caused fibrin to leak out, resulting in fibrinoid necrosis (Figure 2b).

**Figure 3:**
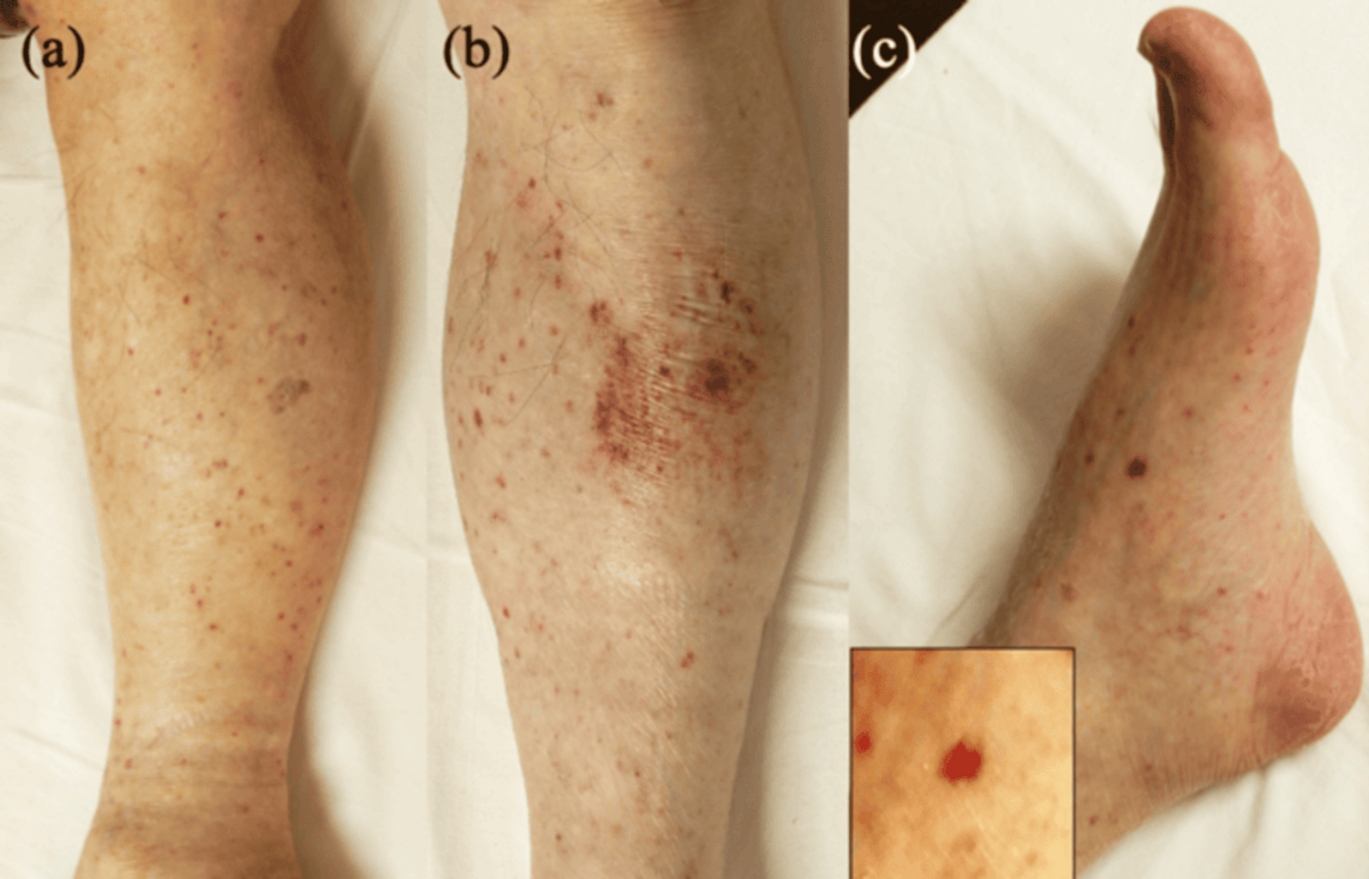
Clinical features, day 10. (a-c) Palpable purpura appeared on the lower extremities and foot soles.

**Figure 4:**
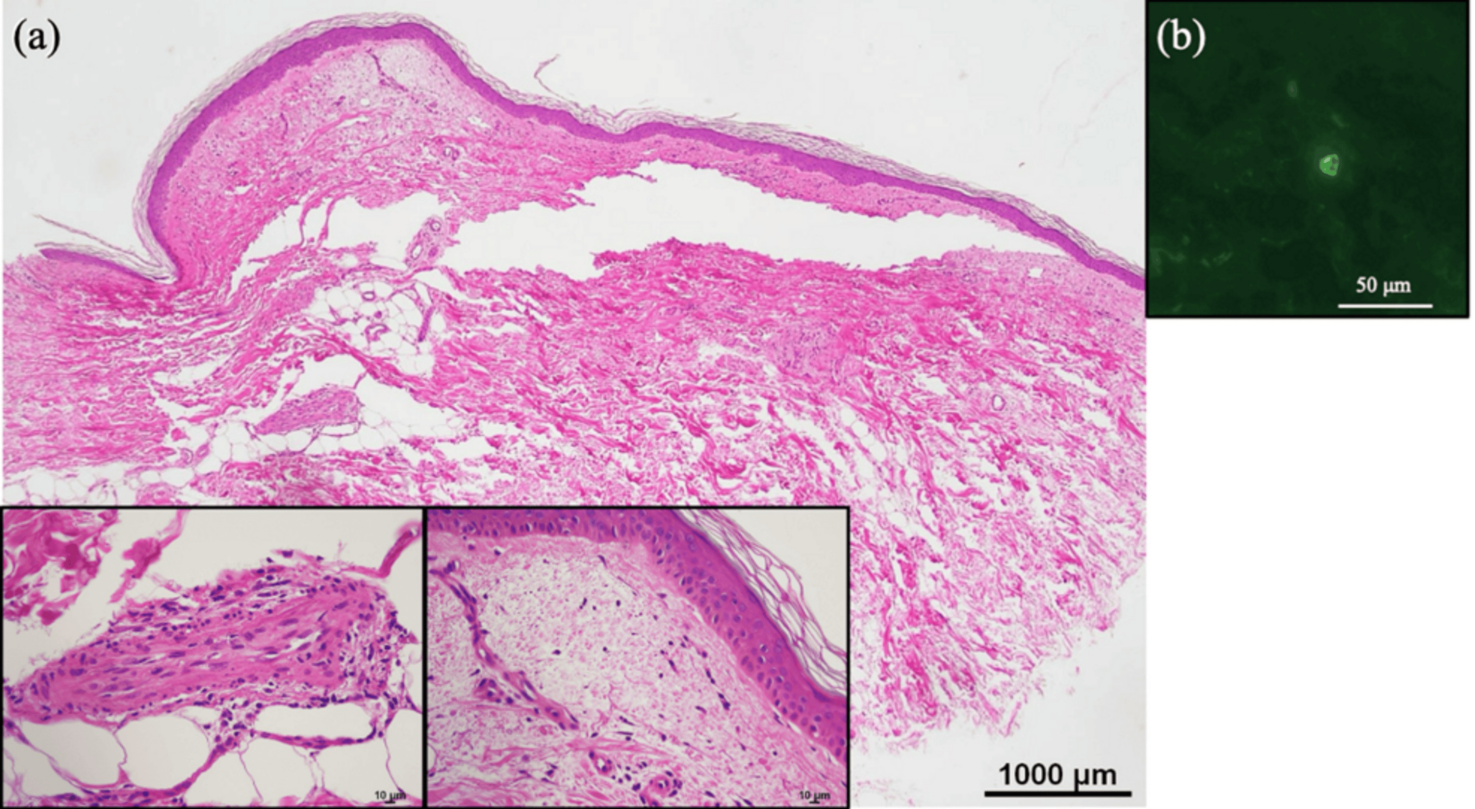
Histopathological findings of palpable purpura on the right lower leg, day 12. (a) Edema and perivascular infiltration of lymphocytes with nuclear debris were observed in the upper dermis. A damaged medium-sized vessel was observed in the deep dermis, with neutrophil infiltration and nuclear debris in the vessel wall. Hematoxylin and eosin staining (original magnification ×40; left and middle lower insert, ×400). (b) Direct immunofluorescence revealed perivascular deposition of IgA in the upper dermis (original magnification ×400).

The patient was treated with intravenous minocycline (200 mg/day, for 2 weeks) and levofloxacin (500 mg/day for the first 11 days). Polymerase chain reaction (PCR) analysis of the whole blood specimen confirmed the presence of *R. japonica* DNA, the causative agent of JSF [1], thereby confirming the diagnosis. As expected, the fever gradually subsided, and the rash resolved within a week; however, some hyperpigmentation remained. Laboratory findings improved to nearly normal levels within two weeks (Table 1). Remarkably, on day 10, the patient developed palpable purpura on the lower extremities and foot soles (Figures 3a-3c). Further laboratory examinations showed elevated IgA (449 mg/dL [normal: 110-410 mg/dL]). Upon biopsy of palpable purpura in the right lower leg at day 12, histopathological examination exhibited edema and lymphocytes infiltration with nuclear debris in the upper dermis, and a damaged medium-sized vessel was observed in the deep dermis, with neutrophil infiltration and nuclear debris in the vessel walls (Figure 4a). DIF assay showed IgA deposition within the small vessel walls in the upper dermis (Figure 4b). Based on these results, we made a diagnosis of palpable purpura secondary to JSF. All purpura resolved within one week without further treatment, and no recurrence was observed over the following six months.

**Table 1:** Blood test results of the present patient. Blood tests revealed leukocytosis, thrombocytopenia, and elevated liver enzymes and CRP. The eosinophil count was deficient from the second day. No renal dysfunction was observed during the course of treatment. Laboratory findings improved to nearly normal levels within two weeks. AST, aspartate aminotransferase; ALT, alanine aminotransferase; CRP, C-reactive protein

Test	Reference Values	Day 1	Day 2	Day 3	Day 5	Day 7	Day 9	Dday 11	Day 14
White blood cells	3,500-9,000 counts/μL	11,920	9,950	10,380	9,100	8,100	9,120	10,450	8,030
Eosinophil	35-510 counts/μL	310	0	0	10	80	230	180	110
Platelet	15.0-35.0 x10^4^ counts/μL	8.1	5.9	4.6	4.3	5.8	9.8	12.6	12.9
AST	5-37 U/L	78	88	84	64	74	51	44	35
ALT	8-42 U/L	30	39	43	39	55	50	41	30
Creatinine	0.6-1.1 mg/dL	0.97	1.01	0.96	0.82	0.72	0.71	0.73	0.77
CRP	<0.5 mg/dL	10.45	18	22.59	11.08	3.87	1.62	1.03	0.67

## Discussion

In this intriguing case, the presence of initial flu-like symptoms, thrombocytopenia, and elevated CRP on blood tests, coupled with the eschar resembling tick bites, led the clinician to diagnose rickettsiosis. To diagnose JSF, it is important to consider the patient's exposure to ticks, i.e., hiking in forests and mountains where deer and wild boars, which can be vectors, are present [8]. However, the patient only took a walk around his house and had no history of outdoor activities or travel [8,9]. This fact suggests that ticks are present even in residential areas and their surroundings. PCR analysis confirmed the presence of *R. japonica*, the causative agent of JSF. It is a gram-negative intracellular bacterium transmitted through tick bites [10]. A standard treatment for the bacterium causing JSF has not yet been established. As with spotted fever group (SFG) of rickettsial infection, the first-line drug is tetracycline. In severe cases, combination therapy involving fluoroquinolone has also been reported to be effective; indeed, a combination of intravenous minocycline and levofloxacin has been successful in the present cases [11,12]. JSF belongs to SFG and causes acute symptoms such as high fever, myalgias, and characteristic rash, specifically characterized by the formation of one or more eschar at the tick bite site accompanied by widespread erythematous rash [1]. Mahara, who first reported JSF, noted that rash turned into petechial spots after three to four days, peaked in a week or 10 days, and disappeared in two weeks [1]. In Rocky Mountain spotted fever, which also belongs to the SFG, erythematous rash occurs in 90% of cases. Furthermore, the rash later becomes petechial spots in 50% of cases [13]. Most rickettsiae, including *R. japonica*, spread through the bloodstream or lymphatic system, targeting vascular endothelial cells, which results in local and systemic vascular injury and inflammation with tissue infiltration of leukocytes and thrombosis [14]. Histopathological studies have documented the presence of leukocytoclastic vasculitis in both the eschar and rash lesions of JSF [2,3]. In fact, the biopsy of the pale erythema on day 1 of this case revealed leukocytoclastic changes in the capillaries of the upper dermis and in medium-sized vessels of the middle to deep dermis (Figures 2a, 2b).

In this case, pulpable purpura similar to IgAV appeared after the rash had disappeared at day 10. We considered the possibility of IgAV associated with Rickettsia infection and performed a biopsy of the purpura at day 12. Finally, in the upper dermis, we found perivascular lymphocytic infiltration and scant nuclear debris, often seen in the late stages of leukocytoclastic vasculitis, along with deeper damaged vasculitis (Figure 4a) [15]. Furthermore, DIF revealed IgA deposition in the epidermal capillaries, suggesting IgAV (Figure 4b).

Various triggers of IgAV have been described, including bacterial and viral infections, vaccines, medications, and solid-organ malignancy [5]. Actually, there are a few reports of IgAV associated with Rickettsia infection [6]. In this case, although the patient was elderly, no serious underlying conditions, including malignant neoplasms, were identified.

While the patient did not take regular oral medications, minocycline and levofloxacin were used for rickettsiosis treatment. However, analyses of two large databases have not reported IgAV onset associated with these two agents [16].

IgAV is a small-vessel vasculitis, characterized by IgA deposition at diseased vessel walls [4]. IgAV begins with an increase in serum IgA and the production of autoantibodies against IgA. This leads to IgA-forming circulating immune complexes, which are deposited on small vessel walls, triggering an inflammatory response. The inflammatory response leads to leukocytoclastic vasculitis [4]. The serum IgA levels are reported to be elevated in 50-70% of patients with IgAV [5], and in this case, there was a slight increase in IgA. This finding supports the interpretation that palpable purpura developed in association with IgA-associated vasculitis.

## Conclusions

We present a case of palpable purpura secondary to JSF. The pathologies of the rickettsial infection rash and IgAV are consistent in that they both exhibit leukocytoclastic vasculitis. However, the mechanism by which IgAV develops following JSF remains unclear. Further accumulation of cases is needed for a detailed analysis of the pathogenesis. This case suggests that JSF may have triggered secondary IgAV, emphasizing the need for clinicians to consider IgAV when palpable purpura occurs after treatment for rickettsiosis.
